# Supplementary material for: Detection of Pneumocystis and Morphological Description of Fungal Distribution and Severity of Infection in Thirty-Six Mammal Species
Source: J Fungi (Basel). 2023 Feb 7;9(2):220. doi: 10.3390/jof9020220 (PMC9960768; doi:10.3390/jof9020220)
Supplement: Supplementary file 1 [file jof-09-00220-s001.zip › jof-2054591-supplementary.pdf]

Figure S1. Alignment of mtSSU rRNA sequences of the present study (NCBI accession numbers [acc. no.] OP738797-OP738809) with published mtSSU rRNA sequences of *P. canis* (NCBI acc. no. MT726216, MT726217), *P. oryctolagi* (NCBI acc. no. NC_060319), *P. carinii* (NCBI acc. no. JX499145), *P. murina* (NCBI acc. no. JX499144), *P.* sp. *ludovicianus* (NCBI acc. no. MT726212), *P.* sp. *macacae* (NCBI acc. no. MT726214), and *P. jirovecii* (NCBI acc. no. JX855936) for confirmation of the affiliation to the genus *Pneumocystis*.

```
                                          10        20        30        40
                                 ....|....|....|....|....|....|....|....|....|..
OP738797_P. sp. bovis            CCACAAAAGTTTCTACAATAGGATGCAAAGATATATTTACAAAGTCA
OP738798_P. sp. ovis             CCACAAAAGTTTCCACAATAGGATGCAAAGATATATTTACAAAGTCA
OP738799_P. sp. caprum           CCACAAAAGTTTCCACAATAGGATGCAAAGATATATTTACAAAGTCA
OP738800_P. sp. rupicaprum       CCACAAAAGTTTCTACAATAGGATGCAAAGATATA--TGTAAAGTCA
OP738801_P. sp. suis             CCACAAAAATTTCTACAATAGGATGCAAAGATGTATGT---AAGTCA
OP738802_P. sp. suis             CCACAAAAATTTCTACAATAGGATGCAAAGATGTATGT---AAGTCA
OP738803_P. canis                CTATAAAAATTTCTACAATAGGATGCAAAGAT--G-----AAAATTT
MT726217_P. canis_Ck1            CTATAAAAATTTCTACAATAGGATGCAAAGAT--G-----AAAATTT
MT726216_P. canis_Ck2            CTATAAAAATTTCTACAATAGGATGCAAAGAT--G-----AAAATCA
OP738804_P. sp. vespertilionis   CCACAAAGATTTCAACAAAGGGATGCAAAAAT--G-----AAAATTA
OP738805_P. sp. soricis          CCACAAAGATTTTTACAAAAGGATGCAAAGAT--G-----AAAGTTG
OP738806_P. leporis              CTACAAAAGTTTCTACAAAGGGAAGCAACAAT--G-----AAAATTA
OP738807_P. oryctolagi           CTGCAAAAGTTTCTACAAAAGGAAGCTAGAAT--G-----AAAATTG
NC_060319_P. oryctolagi          CTGCAAAAGTTTCTACAAAAGGAAGCTAGAAT--G-----AAAATTG
OP738808_P. sp. equi             CCACAAAAATTTCTACAATAGGATGCAAAGATATAATTATTAAGTCA
OP738809_P. carinii              CCACAAATATTTCTACAAAGGGAAGCAAAGATG-------AAAGTCT
JX499145_P. carinii              CCACAAATATTTCTACAAAGGGAAGCAAAGATG-------AAAGTCT
JX499144_P. murina               CCGCAAAAATTTCTACAATGGGAAGCAAAGAT--C-----CAAGTCC
MT726212_P. sp. ludovicianus     CCACAATGATTTCTACAATAGGAAGCAAAGATTTA-----AAAGTCA
MT726214_P. sp. macacae          CTGCAAAAGTTTCTACAATGGGATGCAAAGAT--T-----AAAATCA
JX855936_P. jirovecii            CTGCAAAATTTTCTACAATGGGATGCAATGAT--G-----AAAGTCG

                                    50        60        70        80        90
.                                ..|....|....|....|....|....|....|....|....|....
OP738797_P. sp. bovis            GAGCTAATCCCGGAAAAGAAATTAATGTACAGATAAGAATCTGGAAC
OP738798_P. sp. ovis             GAGCTAATCCCTGAAAAGAAACTAATGTACGGATAAGAATCTGGAAC
OP738799_P. sp. caprum           GAGCTAATCCCTGAAAAGAAAYTAAKGTACGGATAAGAATCKGGAAC
OP738800_P. sp. rupicaprum       GAGCTAATCCCTGAAAAGAAATTAATGTACAGATAAGAATCTGGAAC
OP738801_P. sp. suis             GAGCTAAACCATGAAAGGAAATTAAAGTACGGATAAGAATCTGGAAC
OP738802_P. sp. suis             GAGCTAAACCATGAAAGGAAATTAAAGTACGGATAAGAATCTGGAAC
OP738803_P. canis                GAGCTAATCCCTGAAAAGAAATTTCAGTACAGATAAGAGTCTGAAAC
MT726217_P. canis_Ck1            GAGCTAATCCCTGAAAAGAAATTTCAGTACAGATAAGAGTCTGAAAC
MT726216_P. canis_Ck2            GAGCTAATCCCTGAAAGGAAATTTAAGTACAGATAAGAATCTGAAAC
OP738804_P. sp. vespertilionis   GAGCTAATCCT-TAAATAAAATCATAGTTCAGATAAGAATCTGGAAC
OP738805_P. sp. soricis          GAGCTAATCCT-TAAAGAAAATTTTAGTTCGAATAAGAATCTGGAAC
OP738806_P. leporis              GAGCTAATCCT-CAAAGGAAATTTCAGTACGGATAAGAATCTGGAAC
OP738807_P. oryctolagi           AAGCCAATCCT-CAAAGGAAATTTTAGTCCGAATAAGAATCTGGAAC
NC_060319_P. oryctolagi          AAGCCAATCCT-CAAAGGAAATTTTAGTCCGAATAAGAATCTGGAAC
OP738808_P. sp. equi             GAGCTAATCCAAGAAAAGAAATTAATGTACAGATAAGAATCTGGAAC
OP738809_P. carinii              GAGCTAATCCT-CAAAAGAAATAAAAGTACGGATAAGAATCTGGAAC
JX499145_P. carinii              GAGCTAATCCT-CAAAAGAAATAAAAGTACGGATAAGAATCTGGAAC
JX499144_P. murina               GAGCCAATCCT-TAAAAGAAATTAAAGTACGGATAAGAATCTGGAAC
MT726212_P. sp. ludovicianus     GAGCTAATCCCTGAAAGGAAATATAAGTACGGATAAGAATCTGGAAC
MT726214_P. sp. macacae          GAGCTAATCCT-TAAAAGATATTTTAGTCCGGATAAGTGCCTGGAAC
JX855936_P. jirovecii            GAGCTAATCCCCTAAAAGATTGTTTAGTCCGGATAAGTGCCTGGAAC
```

```
                                          100       110       120       130       140
                                      |....|....|....|....|....|....|....|....|....|.
OP738797_P. sp. bovis                 TCGATTCTTTGAAGAAGGAATTGCTAGTAATCGTCTATCATTACGAG
OP738798_P. sp. ovis                  TCGATTCTTTGAAGAAGGAATTGCTAGTAATCGTCTATCACCATGAG
OP738799_P. sp. caprum                TCGATTCTTTGAAGAAGGAATTGCTAGTAATCGTCTATCACCATGAG
OP738800_P. sp. rupicaprum            TCGATTCTTTGAAGAAGGAATTGCTAGTAATCGTCTATCATCACGAG
OP738801_P. sp. suis                  TCGATTCTTTGAAGAAGGAATTGCTAGTAATCGTCTATCAGCATGAG
OP738802_P. sp. suis                  TCGATTCTTTGAAGAAGGAATTGCTAGTAATCGTCTATCAGCATGAG
OP738803_P. canis                     TCGATTCTTTGAAGATGGAATTGCTAGTAATCGTCTATCAGCATGAG
MT726217_P. canis_Ck1                 TCGATTCTTTGAAGATGGAATTGCTAGTAATCGTCTATCAGCATGAG
MT726216_P. canis_Ck2                 TCGATTCTTTGAAGATGGAATTGCTAGTAATCGTCTATCAGCATGAG
OP738804_P. sp. vespertilionis        TCGATTCTTTGAAGACGGAATTGCTAATAATCGTCTATCAGCATGAG
OP738805_P. sp. soricis               TCGATTCTTTGAAGATGGAATTGCTAGTAATCGTCTATCAGCATGAG
OP738806_P. leporis                   TCGATTCTTTGAAGTTGGAATTGCTAGTAATCGTCTATCAGCAAGAG
OP738807_P. oryctolagi                TCGGTTCTTTGAAGTTGGAATTGCTAGTAATCGTCTATCAGCAAGAG
NC_060319_P. orcytolagi               TCGGTTCTTTGAAGTTGGAATTGCTAGTAATCGTCTATCAGCAAGAG
OP738808_P. sp. equi                  TCGATTCTTTGAAGAAGGAATTGCTAGTAATCGTCTATCACCATGAG
OP738809_P. carinii                   TCGATTCTTTGAAGAAGGAATTGCTAGTAATCGTTCATCATCAAGGA
JX499145_P. carinii                   TCGATTCTTTGAAGAAGGAATTGCTAGTAATCGTTCATCATCAAGGA
JX499144_P. murina                    TCGATTCTTTGAAGAAGGAATTGCTAGTAATCGTTCATCATCAAGGA
MT726212_P. sp. ludovicianus          TCGATTCTTTGAAGCAGGAATTGCTAGTAATCGTCCATCAGCATGGG
MT726214_P. sp. macacae               TCGGCTCTTTGAAGTTGGAATTGCTAGTAATCGTCTATCACCATGAG
JX855936_P. jirovecii                 TCGGCTCTTTGAAGTTGGAATTGCTAGTAATCGTCTATCATCATGAG

                                            150       160       170       180
                                      ...|....|....|....|....|....|....|....|....|...
OP738797_P. sp. bovis                 ACGGTGAAACTATCATCTGTGATGTACTAACTACTCGTCAAGCGCAA
OP738798_P. sp. ovis                  ACGGTGAAACAATCATCTGTGATGTACTAACTACTCGTCAAGCGCAA
OP738799_P. sp. caprum                ACGGTGAAACTATCATCTGTGATGTACTAACTACTCGTCAAGCGCAA
OP738800_P. sp. rupicaprum            ACGGTGAAACTATCATCTGTGATGTACTAACTACTCGTCAAGCGCAA
OP738801_P. sp. suis                  ACGGTGAAACGACCATCTGTGATGTACTAACTACTCGTCAAGCGCAA
OP738802_P. sp. suis                  ACGGTGAAACGACCATCTGTGATGTACTAACTACTCGTCAAGCGCAA
OP738803_P. canis                     ACGGTGAAAGATAAATCTGTGATGTACTAACTACTCGTCAAGCGCGG
MT726217_P. canis_Ck1                 ACGGTGAAAGATAAATCTGTGATGTACTAACTACTCGTCAAGCGCGG
MT726216_P. canis_Ck2                 ACGGTGAAATAAAAATCTGTGATGTACTAACTACTCGTCAAGCGCGG
OP738804_P. sp. vespertilionis        ACGGTGAAACGCCCATCTGTAATGTACTAACTACTCGTCAAGCGCGG
OP738805_P. sp. soricis               ACGGTGAAACAGTCATCTGTGATGTACTAACTACTCGTCAAGCGCAA
OP738806_P. leporis                   ACGGTGAAACTTCTATCTGTGATGTACTAACTACTCGTCAAGCGCGG
OP738807_P. oryctolagi                ACGGTGAAACTTCTATCTGTGATGTACTAACTACTCGTCAAGCGCGG
NC_060319_P. orcytolagi               ACGGTGAAACTTCTATCTGTGATGTACTAACTACTCGTCAAGCGCGG
OP738808_P. sp. equi                  ACGGTGAAACTATCATCTGTGATGTACTAACTACTCGTCAAGCGCAA
OP738809_P. carinii                   ACGGTGAAACGAACATCTGTGATGTACTAACTACTCGTCAAGCGCGA
JX499145_P. carinii                   ACGGTGAAACGAACATCTGTGATGTACTAACTACTCGTCAAGCGCGA
JX499144_P. murina                    ACGGTGAAACAAATATCTGTGATGTACTAACTACTCGTCAAGCGCAA
MT726212_P. sp. ludovicianus          ACGGTGAAACGAACATCTGTGATGTACTAACTACTCGTCAAGCGCAG
MT726214_P. sp. macacae               ACGGTGAATCTACCATCTGTGATGTACTAACTACTCGTCAAGCGCGG
JX855936_P. jirovecii                 ACGGTGAATCTTTTATCTGTGATGTACTAACTACTCGTCAAGCGCGG
```

```
                                    190       200       210       220       230
                                    .|....|....|....|....|....|....|....|....|....|
OP738797_P. sp. bovis               AAATTTC-AGGAAATATAAAGTTTA-TACGTCTATTTCTTAAAGA--
OP738798_P. sp. ovis                AAATTTT-AGGGAATATCAAGTTTA-TACGTCTATTTCTTAAAGA--
OP738799_P. sp. caprum              AAATTTT-AGGRA-TATCAAGTTTA-TACGTCTATTTCTTAAAGA--
OP738800_P. sp. rupicaprum          AAATTAT-AGGAAATATAAAGTTTA-TACGTCTATTTCTTAAAGA--
OP738801_P. sp. suis                AAATTTTTAGGAAATATCAAGTATA-TACGTCTATTTCTTAGAGA--
OP738802_P. sp. suis                AAATTTTTAGGAAATATCAAGTATA-TACGTCTATTTCTTAGAGA--
OP738803_P. canis                   AAATTGATAAGAAATATCAAGTATA-TACGTCTATTTCTTAAAGA--
MT726217_P.canis_Ck1                AAATTGATAAGAAATATCAAGTATA-TACGTCTATTTCTTAAAGA--
MT726216_P.canis_Ck2                AAATTGATAAGAAATATCAAGTTTA-TACGTCTATTTCTTAAAGA--
OP738804_P. sp. vespertilionis      AAATTTATAGGGGATACTAAATTT--TACGACTATTTCCTAGAGA--
OP738805_P. sp. soricis             AAATTTTTAGGGAATACTAAGTTT--TACGTCTATTTCTTAGAGA--
OP738806_P. leporis                 ATATTTATAGGGAATATCAAGCTC--TGCGTCTATTCTCTAGAGA--
OP738807_P. oryctolagi              ATATTTATAGGGAATATCAAGCTC--TGCGTCTATTCTCTAGAGA--
NC_060319_P. oryctolagi             ATATTTATAGGGAATATCAAGCTC--TGCGTCTATTCTCTAGAGA--
OP738808_P. sp. equi                AAATTTT-AGGGAATATCAAGTTTA-TACGTCTATTTCTTAAAGA--
OP738809_P. carinii                 AAATCATTAAGAAGTATCAAGTTGATTGAATTTAATTTCTAAAGAGT
JX499145_P. carinii                 AAATCATTAAGAAGTATCAAGTTGATTGAATTTAATTTCTAAAGAGT
JX499144_P. murina                  AAATCATTAGGGGATATCAAGTTTTGAACGTCTATCCCTTAGAAA--
MT726212_P. sp. ludovicianus        AAATTGTTAGGAAATATCAAGTTTTATACGTCTATTTTCTAGAGA--
MT726214_P. sp. macacae             AAATTTTACAAGAAATCCAAGTTCT-TACGTCAATTTCTTAGAGA--
JX855936_P. jirovecii               AAATTTTTTAAGAAATTCAAGTTCT-TACGTCCATTTCTTGGAGA--

                                       240       250       260       270       280
                                    ....|....|....|....|....|....|....|....|....|..
OP738797_P. sp. bovis               -----------------------------------------------
OP738798_P. sp. ovis                -----------------------------------------------
OP738799_P. sp. caprum              -----------------------------------------------
OP738800_P. sp. rupicaprum          -----------------------------------------------
OP738801_P. sp. suis                -----------------------------------------------
OP738802_P. sp. suis                -----------------------------------------------
OP738803_P. canis                   -----------------------------------------------
MT726217_P.canis_Ck1                -----------------------------------------------
MT726216_P.canis_Ck2                -----------------------------------------------
OP738804_P. sp. vespertilionis      -----------------------------------------------
OP738805_P. sp. soricis             -----------------------------------------------
OP738806_P. leporis                 -----------------------------------------------
OP738807_P. oryctolagi              -----------------------------------------------
NC_060319_P. oryctolagi             -----------------------------------------------
OP738808_P. sp. equi                -----------------------------------------------
OP738809_P. carinii                 TAAAGAATTTAACATCTGTAGAATCAAAGGATTTCAGCGTCTATTTC
JX499145_P. carinii                 TAAAGAATTTAACATCTGTAGAATCAAAGGATTTCAGCGTCTATTTC
JX499144_P. murina                  -----------------------------------------------
MT726212_P. sp. ludovicianus        -----------------------------------------------
MT726214_P. sp. macacae             -----------------------------------------------
JX855936_P. jirovecii               -----------------------------------------------
```

```
                                                    290       300
                                               ..|....|....|....|
OP738797_P. sp. bovis                          -------TTTGTGTTAAG
OP738798_P. sp. ovis                           -------TTTGTGTTAAG
OP738799_P. sp. caprum                         -------TTTGTGTTAAG
OP738800_P. sp. rupicaprum                     -------TTTGTGTTAAG
OP738801_P. sp. suis                           -------TTTGTGTTAAG
OP738802_P. sp. suis                           -------TTTGTGTTAAG
OP738803_P. canis                              -------TCTGTGCTAAG
MT726217_P.canis_Ck1                           -------TCTGTGCTAAG
MT726216_P.canis_Ck2                           -------TCTGTGCTAAG
OP738804_P. sp. vespertilionis                 -------TCTGTGCTAAG
OP738805_P. sp. soricis                        -------TTTGTGCTAAG
OP738806_P. leporis                            -------TTTGTGCTAAG
OP738807_P. oryctolagi                         -------TTTGTGCTAAG
NC_060319_P. oryctolagi                        -------TTTGTGCTAAG
OP738808_P. sp. equi                           -------TTTGTGTTAAG
OP738809_P. carinii                            CTAGAAATTTGTGCTAAG
JX499145_P. carinii                            CTAGAAATTTGTGCTAAG
JX499144_P. murina                             -------TTTGTGCTAAG
MT726212_P. sp. ludovicianus                   -------TTTGTGCTAAG
MT726214_P. sp. macacae                        -------TCTGTGCTAAG
JX855936_P. jirovecii                          -------TCTGTGCTAAG
```
